# Practical Considerations for Accuracy Evaluation in Sensor-Based Machine Learning and Deep Learning

**DOI:** 10.3390/s19163491

**Published:** 2019-08-09

**Authors:** Issam Hammad, Kamal El-Sankary

**Affiliations:** Department of Electrical and Computer Engineering, Dalhousie University, Halifax, NS B3H 4R2, Canada

**Keywords:** ADC, deep learning, edge artificial intelligence (AI), ENOB, machine learning, low power, low quantization, sensor failure, sensor fusion, thermal noise

## Abstract

Accuracy evaluation in machine learning is based on the split of data into a training set and a test set. This critical step is applied to develop machine learning models including models based on sensor data. For sensor-based problems, comparing the accuracy of machine learning models using the train/test split provides only a baseline comparison in ideal situations. Such comparisons won’t consider practical production problems that can impact the inference accuracy such as the sensors’ thermal noise, performance with lower inference quantization, and tolerance to sensor failure. Therefore, this paper proposes a set of practical tests that can be applied when comparing the accuracy of machine learning models for sensor-based problems. First, the impact of the sensors’ thermal noise on the models’ inference accuracy was simulated. Machine learning algorithms have different levels of error resilience to thermal noise, as will be presented. Second, the models’ accuracy using lower inference quantization was compared. Lowering inference quantization leads to lowering the analog-to-digital converter (ADC) resolution which is cost-effective in embedded designs. Moreover, in custom designs, analog-to-digital converters’ (ADCs) effective number of bits (ENOB) is usually lower than the ideal number of bits due to various design factors. Therefore, it is practical to compare models’ accuracy using lower inference quantization. Third, the models’ accuracy tolerance to sensor failure was evaluated and compared. For this study, University of California Irvine (UCI) ‘Daily and Sports Activities’ dataset was used to present these practical tests and their impact on model selection.

## 1. Introduction

The primary objective of solving a problem using machine learning is to obtain a model for generalized predictions. In production, when deploying a pretrained machine learning model for inference, it should be expected that this model will perform predictions with an accuracy close to the achieved test accuracy during prototyping. Large deviations between the reported test accuracy and the actual accuracy in production can be a serious design problem. Therefore, the test accuracy should consider any practical aspects or variables that don’t necessarily exist in the development but can occur in production. In the early stages of machine learning research, many papers reported the training accuracy as the model’s prediction accuracy. This practice doesn’t truly reflect how the model can generalize to new data, instead, it reflects how good the model can fit the training set, which often can be a case of overfitting. Achieving a low generalization error that characterizes prediction performance and avoids overfitting and underfitting is discussed with more details in [1] and [2]. One of the early research papers that emphasized the importance of using a separate test set for model evaluation is [3]. Nowadays, machine learning model accuracy evaluation is performed by splitting the available data into training, cross-validation, and test sets. The cross-validation set is usually used to tune the models’ hyperparameters, while the test set determines the prediction accuracy of the model. In many instances, the dataset is split into training and testing sets only, where the test set is used for cross-validation and to determine the prediction accuracy.

Using the train/test split is very common and is considered an acceptable practice in machine learning research today. Many train/test split techniques are used in the literature such as k-fold cross-validation and Monte Carlo cross-validation (MCCV). These techniques were used in different sensor-based machine learning research problems such as [4,5,6,7,8]. These papers built different machine learning models for sensor-based problems and compared their accuracy using the common train/test split. It is common in machine learning to build multiple models using different algorithms for one problem, then determine the best model for that problem based on the top achieved test set accuracy. However, for sensor-based machine learning problems, models’ accuracy and suitability comparison should take into consideration practical factors that can occur in production.

Presenting these practical production considerations and their impact on model selection in sensor-based problems is the focus of this paper. These presented practical tests include the impact of thermal noise on the models’ inference accuracy, models’ accuracy tolerance to lower inference quantization, and model tolerance to sensor failure. The next section will provide a detailed background on these practical tests and their purpose. The paper will demonstrate that when considering these practical production problems, the decision regarding the appropriate machine learning model for a problem can be majorly impacted.

For this study, the University of California Irvine (UCI) ‘Daily and Sports Activities’ dataset was employed. The dataset was constructed by [4] and is posted on the online repository [9]. The dataset contains sensor readings from accelerometers, gyroscopes, and magnetometers which correspond to a number of physical activities performed by different participants.

This paper is divided as follows, Section 2 provides a background on the proposed practical accuracy tests and their role in model selection. Section 3 describes the details and structure of the dataset used in this study. Section 4 presents the achieved baseline accuracies for various machine learning models using k-fold train/test split. In Section 5 the experimental results for the proposed practical tests are demonstrated. Finally, Section 6 presents the research conclusions.

## 2. Background on the Proposed Practical Accuracy Tests

In sensor-based machine learning, accuracy evaluation using the train/test split can be sufficient to determine if the model works and to provide a baseline accuracy evaluation only. To build a production-ready machine learning model for sensor-based problems, accuracy evaluation should consider practical aspects such as: Studying the impact of thermal noise on the inference accuracy, finding the adequate level of inference quantization, and evaluating model accuracy tolerance to sensors failure. Sensors have thermal noise, which varies even between sensors of the same model. On the other hand, machine learning datasets are constructed using a fixed number of sensors in a specific environment. Therefore, the impact of this independent thermal noise is not reflected in the basic train/test split accuracy evaluations.

According to [10], it can be reasonably assumed that each accelerometer creates its own independent thermal noise. Based on [11], an accelerometer’s thermal noise can be modeled as an additive zero-mean Gaussian noise. The presented thermal noise simulation in this paper will demonstrate that machine learning models can have significantly different levels of accuracy loss due to thermal noise. Another practical production aspect is finding the adequate inference quantization level and the best model for this level of quantization. With the rise of edge artificial intelligence (AI) technology, pretrained machine learning models are deployed directly on embedded hardware, which is often low-power. Lowering the inference quantization can reduce the hardware cost of the analog-to-digital converter (ADC) and other digital signal processing (DSP) components. Speed–power–accuracy trade-offs in high-speed complementary metal-oxide-semiconductor (CMOS) ADCs are detailed in [12]. Based on [12], higher bit accuracy requires larger devices that result in lower speed and/or higher power consumption. Evaluating models’ inference accuracy using different quantization levels will determine the adequate level and will impact the decision on the model selection. Also, it is known that the ADC effective number of bits (ENOB) is usually lower than the ideal number of bits [13]. Hence, it will be critical to evaluate models’ tolerance with lower inference quantization to simulate this practical ADC problem. A final aspect to consider is the impact of sensor failure on machine learning inference accuracy. Models with more tolerance to sensor failure might be favorable for production. Also, redundancy or other failure mitigation solutions can be applied for sensors with greater impact on the accuracy. The simulation for all these practical problems is presented in Section 5.

## 3. Dataset Details and Simulation Tools

**Figure 1:**
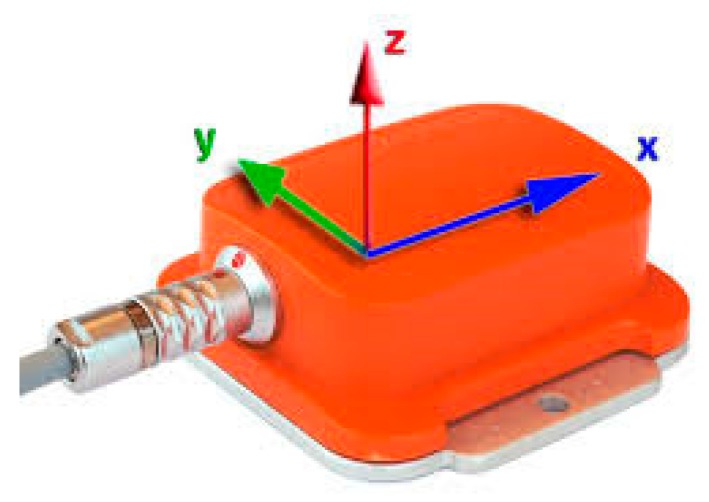
Xsens MTx 3-DOF (degrees of freedom) orientation tracker (photo from [14]).

For this study, the UCI ‘Daily and Sports Activities’ dataset was employed. The dataset was published by [4] and is posted on the UCI online repository [9]. This dataset was constructed by using five Xsens MTx 3-DOF (degrees of freedom) orientation trackers. Figure 1 illustrates the used orientation tracker which was developed by Xsens Technologies. A total of 8 participants, 4 males and 4 females aged 20–30 contributed in the construction of the dataset [9]. For each participant, the orientation trackers were placed on the torso (tracker #1), the right arm (tracker #2), the left arm (tracker #3), the right leg (tracker #4), and the left leg (tracker #5). Data from these orientation trackers were captured during 19 different physical activities that the participants performed. These 19 activities were [4]: (1) sitting, (2) standing, (3) lying on back, (4) lying on right side, (5) ascending stairs, (6) descending stairs, (7) standing in an elevator still, (8) moving around in an elevator, (9) walking in a parking lot, (10) walking on a treadmill in flat with a speed of 4 km/h, (11) walking on a treadmill with 15° incline with a speed of 4 km/h,(12) running on a treadmill with a speed of 8 km/h, (13) exercising on a stepper, (14) exercising on a cross trainer, (15) cycling on an exercise bike in horizontal position, (16) cycling on an exercise bike in vertical positions, (17) rowing, (18) jumping, and (19) playing basketball. This dataset presents a classification problem, where the input is constructed from the readings of the 5 orientation trackers, while the output represents one of the 19 physical activity classes. Based on the Xsens MTx manual [14], each orientation tracker contains a 3D accelerometer, 3D gyroscope, and a 3D magnetometer. For analog to digital conversion, 16 bits ADC is used [14]. This configuration resulted in having 9 sensor readings per tracker and a total of 45 sensor readings per record. The dataset was constructed using a sampling frequency of 25 Hz with 5 s representing each labeled instance. Therefore, 125 records, with 45 sensor readings each, construct one labeled instance. Accordingly, each instance had 5625 attributes. The entire dataset contained 9120 instances. The dataset was balanced as there were 60 instances for each activity per participant.

This dataset was selected to study the proposed practical considerations for accuracy as it provides the raw data readings of the sensors. Also, it specifies that Xsens MTx orientation trackers [14] were used, which is unlike many machine learning datasets that don’t include information on the hardware used. Providing the raw data and the hardware details facilitated the presented theory and simulations in this paper. Many research papers have proposed different machine learning models for this dataset, including the dataset publisher in [4,5]. In addition to that, several other papers proposed additional machine learning models and techniques such as [15,16,17]. The research goal was to demonstrate how the baseline accuracy achieved by the train/test split can be significantly impacted when considering the proposed practical tests such as the thermal noise impact, the impact of lower inference quantization, and model’s accuracy tolerance sensor failure. Model development and the simulation of the practical tests were implemented using the popular machine learning and data analysis python libraries: Keras [18], Sci-kit-learn [19], and NumPy.

## 4. Baseline Accuracy

Prior to presenting the proposed practical accuracy tests, baseline accuracies should be established using the basic train/test split. These baseline accuracies will be used later to demonstrate the impact of the proposed practical on the models’ accuracy. Several machine learning models were trained as part of this research work on the dataset [9] using two different input sizes. One, by applying dimensionality reduction using principle component analysis (PCA), while the other by using the original raw data without any reduction. The number of attributes for each instance was reduced from 5626 to 30 when PCA was applied. Several popular machine learning algorithms were employed in this study. For each algorithm, multiple models with different hyperparameter settings were tested.

Table 1 lists the achieved test accuracy for the top model using each listed algorithm. Selecting these top models was achieved by iterating over multiple possible models representing different configurations for each algorithm, then performing the training and the cross-validation. The test accuracy results were logged and filtered, then the models with top test accuracies were trained again for confirmation. The purpose of the models’ accuracies listed in Table 1 is to act as a reference when studying the impact of thermal noise, low quantization, and sensor failure on the overall accuracy of each model. The paper uses its own baseline model for the purpose of presenting the practical accuracy with the same training setting and testbench when using the baseline models for comparison. Therefore, any drop in the accuracy will be due to introducing the proposed practical tests. This determines how resilient the accuracy of these models is when considering these practical considerations. Table 1 accuracies were obtained by applying k-fold cross-validation with k = 10. The data were divided as 90% for training and 10% for testing. This resulted in having 912 test instances in each k-fold iteration. As can be seen in Table 1, the deep neural network (DNN) model without PCA achieved the top accuracy, while the random forest classifier (RFC) without PCA achieved the second-best accuracy. The accuracy difference between the two models was 0.3%, which is considered very minimal and can be negligible. Additionally, the results indicated that using PCA increased the accuracy of some models, while for other models it had a negative impact. The DNN model was built using the popular deep learning [20] platform Keras [18], while the remaining machine learning models were built using scikit-learn [19]. Table 1 lists the DNN test accuracy for the best deep learning model. This DNN has five dense layers with the following sizes: Layer #1 (512 neurons), layer #2 (128 neurons), layer #3 (128 neurons), layer #4 (64 neurons), layer #5 (19 neurons). Layers 1–4 used ReLU activation function, while the output layer (layer #5) used a softmax activation function. Dropout was used for regularization and batch normalization was used in all the layers. For RFC, the model used 250 trees. For k-nearest neighbors (KNN) model, it was determined that the algorithm performs the best with eight neighbors. For the remaining algorithms, it was determined that the default scikit-learn settings achieved the best accuracy for each model.

In the next section, the simulation results for the proposed practical accuracy tests will be presented. In order to have a manageable number of simulations that can be clearly compared, one model per algorithm is used to present the practical accuracy simulations in this paper. The selection was done based on the model with higher accuracy, either including PCA or excluding it. An exception was applied in the cases of RFC and DTC as the test accuracies including and excluding PCA were extremely close, therefore, both models were included. This allows for an evaluation of the impact of dimensionality reduction when applying the proposed practical tests as well.

## 5. Experimental Results

This section presents the experimental results for the proposed practical accuracy tests. In the first set of tests, models’ inference accuracy loss due to thermal noise was evaluated. This was achieved by simulating different levels of signal-to-noise ratio (SNR) for possible sensors’ thermal noise. The results demonstrate that even though different machine learning models can have similar baseline test accuracies, their tolerance to the thermal noise can vary significantly. Therefore, the selection of the appropriate machine learning model should consider the expected levels of SNR that the sensors have. The second set of tests evaluate models’ accuracy tolerance to different quantization levels applied to the test set. The aim of these tests was to determine the adequate inference quantization level and the model that can achieve the highest accuracy at this level. Accordingly, for any custom design, the accuracy loss with lower inference quantization and the complexity of the model can be balanced against the required ADC resolution and the cost of any DSP components. Lower inference quantization also simulated the ADC ENOB problem. Models can have close baseline accuracies using high inference quantization; however, the accuracy loss with lower inference quantization can vary significantly from model to model. Additionally, a simulation for the inference accuracy with lower training quantization levels is presented. This determined whether or not a lower level training quantization is required to achieve better lower inference quantization accuracy. The third set of tests presents models’ accuracy tolerance to sensor failure. Models with higher tolerance to sensor failure might be favorable for embedded designs. Such analysis will enable designers to evaluate the impact of a failure in a specific sensor or tracker on the models’ accuracy. Therefore, more design constraints or a failure mitigation solution can be applied to sensors with higher impact on the accuracy.

The experimental results for the proposed practical accuracy tests are presented in the next three subsections. In Section 5.1, the simulation for the thermal noise impact on the accuracy is presented. Section 5.2 demonstrates the impact of low inference quantization on the accuracy, while in Section 5.3, the simulation results for the impact of sensor failure on the accuracy are presented.

### 5.1. Thermal Noise Simulation

Thermal noise, or Johnson–Nyquist noise, exists in all electrical circuits and it is caused by the random thermal motion of electrons. Thermal noise is approximately white with a Gaussian probability density function (PDF) amplitude [21]. Thermal noise is independent for each component, where each component has its own thermal noise. For example, in accelerometers, according to [10], each accelerometer has its own independent thermal noise. Also, based on [11], the thermal noise in accelerometers can be modeled as an additive zero-mean Gaussian noise. For gyroscopes, according to [22], the thermal noise in the gyroscope can be also modeled as Gaussian zero-mean independent noise. Even though the original dataset readings contain thermal noise as part of the reading, this noise is specific to the sensors used during the capture of the original dataset at the time of capture. During training, these captured sensor readings with this noise included will establish the foundations of any machine learning model. Therefore, the impact of thermal noise due to changes in the components, the timing, or the environment will impact the inference accuracy and not the training accuracy.

Examples can be provided from the literature for thermal noise SNR levels. For instance, in [23], a low noise accelerometer which can be used in medical applications can have a threshold of 20 dB SNR. Based on [10], SNR of 0 dB or above for 2D accelerometers is considered good. In [24], an example is provided for search coil magnetometer with a thermal noise of 23 dB. Based on this information, a simulation for various levels of SNR resulting from adding zero-mean Gaussian noise is practical and realistic. For this simulation, various ranges of SNR were simulated, starting with 40 dB and going to 0 dB, with a 5 dB reduction between the test cases. These SNR values were simulated by adding a randomly generated zero-mean Gaussian noise with specific power to the test set.

**Figure 2:**
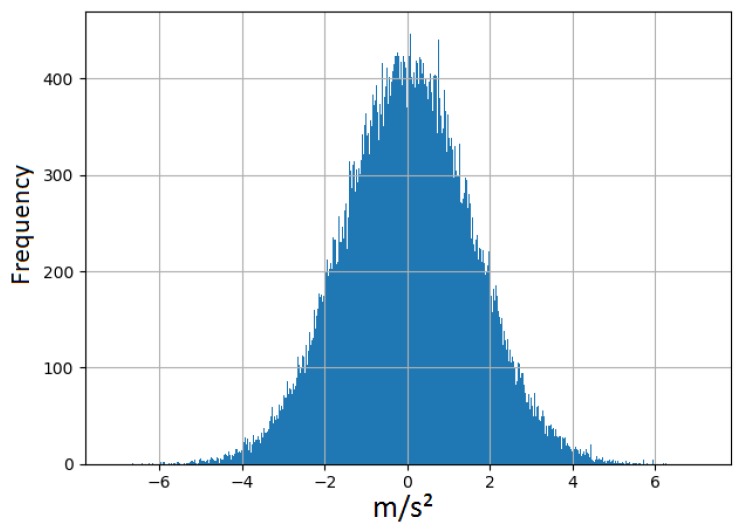
A histogram for a thermal noise sample added to one accelerometer axis in all test instances.

**Figure 3:**
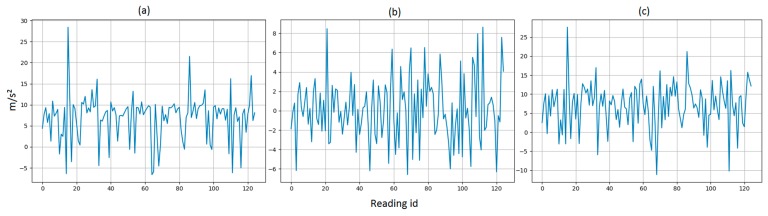
A sample for thermal noise simulation for one accelerometer axis in one instance with signal-to-noise ratio (SNR) of 5 dB. (**a**) Original sensor readings. (**b**) Added white noise. (**c**) New values with SNR = 5 dB.

Table 2 demonstrates the accuracy for machine learning models at each specific SNR value. The listed accuracy for each test case in Table 2 was obtained by averaging 25 random simulations of thermal noise for each k-fold. Hence, each listed accuracy resulted from averaging a total of 250 iterations. Figure 2 illustrates a histogram for a sample thermal noise distribution which was added to one accelerometer axis during one test iteration. Figure 2 was constructed from 11,400 points, resulting from adding the noise to one accelerometer axis for all 912 test instances. As previously mentioned, each instance represents a period of 5 s with a sampling frequency of 25 Hz. Figure 3 illustrates an example of thermal noise simulation for one accelerometer axis in one test instance. As can be seen from Table 2, the accuracy tolerance for models significantly varied due to thermal noise. For example, when comparing DNN and RFC, both models had very close baseline accuracies with a difference of 0.3% only. Therefore, a developer might prefer to deploy RFC over DNN based on certain design or performance aspects. However, when considering the models’ tolerance to thermal noise, it becomes clear that DNN is superior over RFC. As an example, when considering 20 dB SNR level, the accuracy difference between these two models changed from 0.3% to 4.36%, which is significant. Another example can be seen when comparing KNN + PCA and RFC.

**Figure 4:**
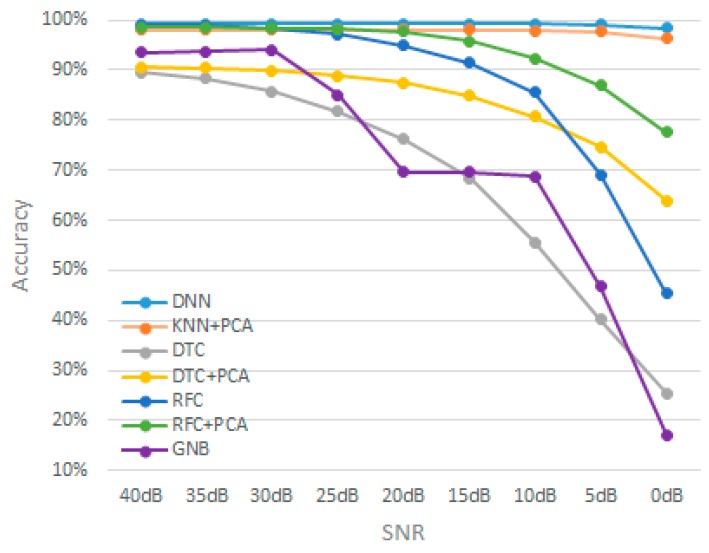
Accuracy trend for machine learning models with the increase of thermal noise power.

According to the baseline accuracies, RFC is better than KNN + PCA. However, at 25 dB SNR, KNN + PCA surpassed the RFC model and the accuracy gap increased accuracy with higher noise. Figure 4 shows the models’ accuracy trend with the increase of thermal noise power. This analysis will enable designers to choose the appropriate model for their sensor-based machine learning problem according to the expected level of thermal noise. Also, the Figure provides a trade-off between the feasibility of using sensors with higher thermal noise, which has lower cost including the power and the willingness to have machine learning designs with lower accuracy.

### 5.2. Quantization Levels Simulation

Lowering the inference quantization level can reduce the costs for possible embedded/edge AI implementations for the machine learning model. Using lower inference quantization will lower the resolution for the used ADC and the other DSP components. This will be cost-efficient in embedded implementations as lower resolution ADCs have lower power and higher bandwidth [12]. In addition to reducing the ADC resolution during inference, it is known that ADCs ENOB is usually lower than the ideal ADC bits due to the quantization error [13]. As an example, the 8 bits ADC in [13] achieved 6.6 ENOB. Therefore, applying inference on lower resolutions can simulate the ADC ENOB as well. The ADC itself, can’t fully ensure the accuracy of results. Many factors including voltage reference, PCB layout, I/O switching, and analog source impedance can affect the overall ADC accuracy [25].

**Figure 5:**
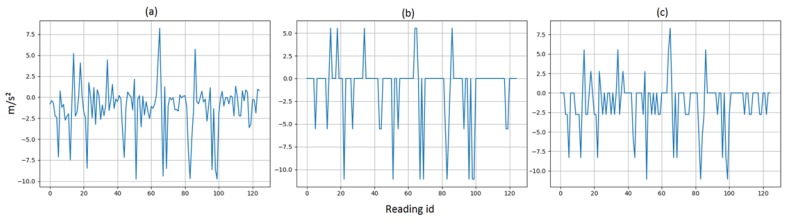
A sample for low quantization simulation for one accelerometer axis in one instance. (**a**) Original sensors readings with 16 bits quantization. (**b**) 5 bits quantization (**c**) 6 bits quantization.

**Figure 6:**
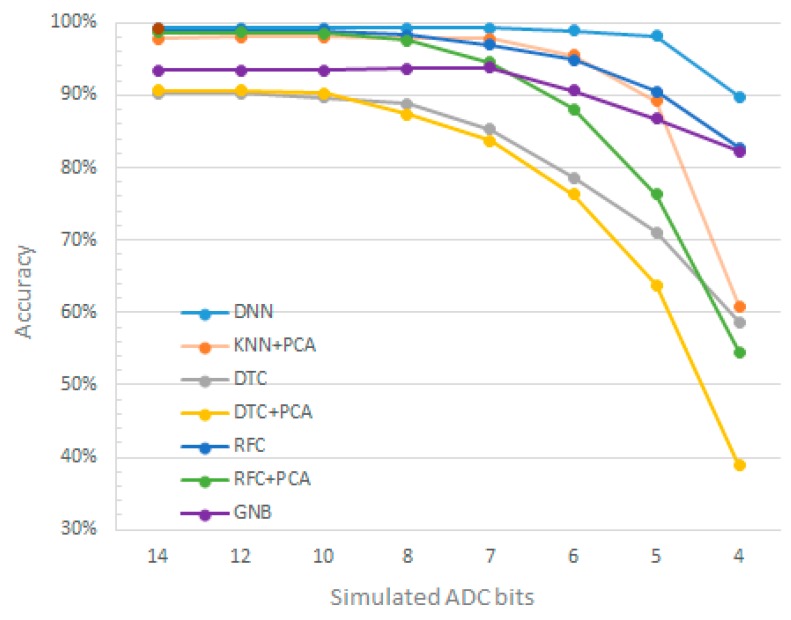
Accuracy trend in machine learning models with lower inference quantization.

The proposed practical tests for lower inference quantization will answer the following questions. First, what is the accuracy loss for each model when lowering the inference quantization? This should allow the designer to determine the trade-off between the possible ADC resolutions and accuracy loss in each model. It will also provide a practical accuracy evaluation when considering the ADC ENOB problem. Second, which model achieves the highest accuracy at the required quantization level? Third, is there a performance difference between training with high quantization, then applying inference with lower quantization vs. implementing both the training and the inference with lower quantization? Table 3 lists the models’ inference accuracies using a range of simulated resolutions for the ADC. For the training stage, the quantization used the original dataset levels [9] at 16 bits, and the lower quantization was applied on the test set. Figure 5 demonstrates the simulation for 5 bits (32 levels) and 6 bits (64 levels) ADC for one accelerometer axis in one instance. In Figure 6 models’ accuracy trend with lower inference quantization is illustrated.

As can be seen from Table 3 and Figure 6, DNN has a very high tolerance to lower quantization. For the DNN model, lowering the inference quantization from 16 bits to 6 bits resulted in an accuracy loss of only 0.36%. DNN also achieved higher tolerance to thermal noise as per the previous section. However, for the other models, the accuracy loss trend with lower quantization was different compared to the thermal noise impact, which was simulated in the previous section. This is due to the fact that the nature of these problems is different. The thermal noise is uncorrelated [26], while the quantization noise is partly correlated [27]. For example, the GNB model was intolerant to thermal noise, while it tolerated lower quantization relatively well compared to the other models. KNN + PCA showed more resilience in the case of thermal noise. On the other hand, RFC showed more resilience in the case of lower quantization. Additionally, it can be seen from Table 3 that models with dimensionality reduction using PCA have higher accuracy loss with lower quantization compared to models that excluded PCA. This can be seen when comparing DTC vs. DTC + PCA and RFC vs. RFC + PCA.

Lowering the resolution of an ADC is directly proportional to its energy. An ideal N-bit ADC SNR is calculated as [28]:

SNR = 6.02 N + 1.76 dB.(1)

Using Murmann’s popular ADC survey [29]. A proportional relationship can be established between an ADC signal to noise and distortion ratio (SNDR) and its energy in picojoules (pJ). Hence, the number of bits in an ADC is directly proportional to its energy. Therefore, tolerating a lower inference quantization leads to a reduction in the required conversion energy for the ADC in any custom design. For embedded and low power applications, lowering quantization is primarily beneficial for inference and not for training. For such applications, it is expected that training is done using powerful computers, then machine learning models can be deployed on chip for inference. Nevertheless, some models require training with lower quantization to achieve better accuracies with low inference quantization. Table 4 lists the achieved inference accuracy when applying lower quantization to the training phase as well. As can be seen, the accuracies were different compared to Table 3. For example, DNN with 5 bits inference quantization achieved an accuracy of 98.11%. However, if the 5 bits quantization is applied for both training and inference, the accuracy drops to 89.29%. Like DNN, GNB performed better when lower quantization was applied during the inference level only. On the other hand, DTC and RFC performed better when the lower quantization was applied for training as well. In summary, for low inference quantization, it will be critical to determine if low quantization is required during the training stage or not. This will vary from model to model, as can be seen. It is advisable in such cases to capture the dataset using high resolution ADCs, then based on the selected model, decide if the training set quantization level should be lowered or not.

### 5.3. Impact of Sensor Failure on the Accuracy

Sensor failure is common and can occur at any time. In [30], statistics regarding sensor failures in smart homes are presented. Based on [30], the most common failure modes for sensors in smart homes are: Data link loss, either wired or wireless; dead battery or the loss of power’ and loss of internet connection. Also, sensors can fail due to different mechanical issues. Hence, a practical machine learning design could require a certain level of tolerance to sensor failure during inference. Models with higher accuracy tolerance to sensor failure might be preferable for production. Also, such analysis will allow designers to apply a failure mitigation solution for sensors with greater impact on the accuracy. This includes using hardware redundancy or using higher quality sensors. Table 5 lists the accuracy for each model given a device in a tracker has failed. The table assumes one device (accelerometer, gyroscope, or magnetometer) with all 3-dimensional sensors has failed in one of the trackers. The listed accuracies are based on averaging all possible failure scenarios for each device type. Device failure is simulated by setting the device 3-dimensional readings to zero in the test set.

As can be seen from Table 5, the gyroscope has the least influence on the accuracy in all the models. Therefore, for production, using a lower quality gyroscope might be acceptable. On the other hand, depending on the model, the accelerometer, the magnetometer, or both, have a great impact on the accuracy. For example, the failure of a magnetometer had the greatest impact on the DNN’s accuracy. However, the case was different for models with PCA, where the magnetometer had significantly less impact compared to the accelerometer. Therefore, based on the model, applying a failure mitigation solution for sensors with the greatest impact on the accuracy could be an option. This will ensure that the final design in production has a greater accuracy tolerance towards such failures.

Table 6 lists the inference accuracy for each model, assuming an entire tracker with all its nine sensors has failed. The same analogy that was applied to Table 5 can be applied to Table 6. Based on the selected model, one or more trackers with the greatest impact on the accuracy can have a failure mitigation solution.

## 6. Conclusions

This paper proposed a set of practical tests that can be applied to compare the accuracy of sensor-based machine learning models. To select an appropriate machine learning model for production in a sensor-based application, several practical aspects should be considered beyond the basic train/test accuracy comparison. Using the UCI ‘Daily and Sports Activities’ dataset, these practical aspects were presented. First, in production, sensors’ independent thermal noise will impact the models’ inference accuracy negatively. Therefore, practical evaluation of the models’ accuracy should consider the expected level of sensor’s thermal noise. By simulating different levels of SNR, it was demonstrated that models’ accuracy tolerance to thermal noise can vary significantly from model to model. Consequently, the decision on the appropriate model for deployment in production could be impacted. As an example, at 20 dB SNR, DNN had an average accuracy loss of 0.02%, while RFC had an average accuracy loss of 4.08%. Both models had a close baseline train/test accuracy with only 0.3% difference. The second presented practical tests aimed to find the adequate inference quantization level. For embedded AI applications, lowering inference quantization leads to lowering the required ADC resolution. Additionally, ADC ENOB is usually lower than its ideal number of bits. Accordingly, a simulation of lower inference quantization addressed both problems. Simulation results showed that the models’ accuracy tolerance to lower inference quantization can vary significantly. DNN had the lowest accuracy loss using low inference quantization levels. DNN achieved an accuracy of 98.11% with only 5 bits quantization, which is only a 1.15% of accuracy loss compared to 16 bits quantization. The simulation results also showed that the models’ dimensionality reduction using PCA were intolerant to lower inference quantization levels. Additionally, some models required lower training quantization levels to achieve better accuracies using lower inference quantization levels. This could be seen in the cases of the RFC and the DTC models, which were in contrast with the DNN and the GNB models. Therefore, to lower the ADC resolution, lower quantization should either be applied during both the training stage and inference stage or during inference only. This varies based on the model. Finally, the impact of sensors failure on the models’ accuracy was presented. The proposed sensor failure tests can help designers in selecting models with higher accuracy tolerance to such failures for deployment in production. Also, it will allow designers to apply failure mitigation solutions on sensors with greater impact on the model’s accuracy. While the UCI ‘Daily and Sports Activities’ dataset was used in this paper, the proposed practical accuracy tests are generic and are not limited to this dataset. The same practical accuracy tests can apply to any sensor-based machine learning problem.

## Figures and Tables

**Table 1:** Baseline test accuracies using k-fold (k = 10).

Algorithm	Train/Test Sample Size	Test Accuracy without PCA	Test Accuracy with PCA
Deep Neural Network (DNN)	8208/912	99.26%	97.87%
K-Nearest Neighbors (KNN)	8208/912	78.34%	98.12%
Decision Tree Classifier (DTC)	8208/912	90.30%	90.72%
Random Forest Classifier (RFC)	8208/912	98.96%	98.65%
Gaussian Naïve Bayes (GNB)	8208/912	93.49%	78.55%

**Table 2:** Average inference accuracy with simulated thermal noise.

SNR	Machine Learning Model						
	DNN	KNN + PCA	DTC	DTC + PCA	RFC	RFC + PCA	GNB
Baseline	99.26%	98.12%	90.30%	90.72%	98.96%	98.65%	93.49%
40 dB	99.28%	98.11%	89.62%	90.54%	98.93%	98.59%	93.34%
35 dB	99.25%	97.97%	88.30%	90.47%	98.84%	98.51%	93.28%
30 dB	99.25%	97.98%	85.70%	89.89%	98.35%	98.44%	93.03%
25 dB	99.27%	98.02%	81.69%	88.90%	97.08%	98.24%	85.06%
20 dB	99.24%	98.03%	76.28%	87.53%	94.88%	97.60%	69.61%
15 dB	99.25%	98.01%	68.33%	84.79%	91.51%	95.74%	69.60%
10 dB	99.24%	97.82%	55.65%	80.77%	85.55%	92.35%	68.81%
5 dB	99.11%	97.73%	40.13%	74.56%	69.12%	86.90%	46.82%
0 dB	98.43%	96.37%	25.46%	63.98%	45.40%	77.61%	17.07%

**Table 3:** Average inference accuracy using low resolution inference accuracy.

Resolution	Machine Learning Model						
	DNN	KNN + PCA	DTC	DTC + PCA	RFC	RFC + PCA	GNB
16 bits[baseline]	99.26%	98.12%	90.30%	90.72%	98.96%	98.65%	93.49%
14 bits	99.25%	97.95%	90.28%	90.68%	98.95%	98.61%	93.40%
12 bits	99.25%	98.02%	90.23%	90.64%	98.93%	98.61%	93.47%
10 bits	99.25%	97.99%	89.62%	90.31%	98.89%	98.56%	93.44%
8 bits	99.20%	97.93%	88.80%	87.30%	98.33%	97.50%	93.72%
7 bits	99.20%	97.74%	85.33%	83.68%	96.94%	94.53%	93.74%
6 bits	98.90%	95.48%	78.63%	76.33%	94.89%	88.11%	90.65%
5 bits	98.11%	89.12%	71.01%	63.81%	90.51%	76.29%	86.69%
4 bits	89.74%	60.91%	58.62%	38.89%	82.71%	54.52%	82.26%

**Table 4:** Average inference accuracies using lower resolution quantization applied to training and testing.

Model	Simulated ADC Bits				
	8 bits	7 bits	6 bits	5 bits	4 bits
DNN	99.19%	98.72%	98.54%	89.29%	81.87%
KNN + PCA	98.03%	95.29%	91.56%	72.48%	51.65%
DTC	89.06%	86.32%	87.41%	79.96%	75.35%
DTC + PCA	86.29%	81.91%	79.17%	50.88%	36.73%
RFC	98.80%	97.48%	97.70%	92.77%	89.81%
RFC + PCA	96.24%	94.59%	87.91%	67.73%	41.31%
GNB	85.62%	82.55%	81.67%	54.04%	32.11%

**Table 5:** Inference accuracy with a device failure in one tracker.

Model	Failed Device		
	Accelerometer	Gyroscope	Magnetometer
DNN	93.75%	98.81%	83.92%
KNN + PCA	64.42%	94.77%	94.74%
DTC	63.98%	90.28%	66.26%
DTC + PCA	30.46%	90.72%	90.48%
RFC	87.55%	98.82%	82.58%
RFC + PCA	41.38%	98.26%	96.26%
GNB	76.87%	92.79%	86.08%

**Table 6:** Inference accuracy with one tracker failure.

Model	Failed Tracker				
	#1	#2	#3	#4	#5
DNN	74.29%	86.15%	72.59%	74.69%	68.65%
KNN + PCA	78.08%	48.69%	51.07%	71.53%	72.16%
DTC	38.6%	66.89%	55.27%	64.37%	16.15%
DTC + PCA	27.89%	33.43%	30.63%	31.56%	28.9%
RFC	57.73%	82.69%	73.26%	88.59%	39.62%
RFC + PCA	42.98%	40.48%	38.88%	43.84%	40.25%
GNB	57.21%	83%	67.55%	63.45%	64.5%
